# Analysis of risk factors for acute cerebral infarction in patients with intracranial tuberculosis

**DOI:** 10.3389/fneur.2024.1493715

**Published:** 2025-01-07

**Authors:** Xiao-Shan Huang, Xiao-Wei Qiu, An-Long Wang, Fei He, Yi-Jing Wang

**Affiliations:** ^1^Department of Radiology, The Second Affiliated Hospital of Zhejiang Chinese Medical University, Hangzhou, China; ^2^Department of Radiology, Hangzhou Red Cross Hospital, Hangzhou, China; ^3^Respiratory Department, Hangzhou Red Cross Hospital, Hangzhou, China

**Keywords:** intracranial tuberculosis, cerebral infarction, risk factors, MRI, arteritis

## Abstract

**Objective:**

Acute cerebral infarction is a common complication of intracranial tuberculosis (TB), causing irreversible damage to brain tissue and significantly affecting patient prognosis. This study aims to explore the risk factors associated with acute cerebral infarction in patients with intracranial tuberculosis.

**Methods:**

We retrospectively analyzed data from eligible intracranial TB patients treated at our hospital between January 2020 and March 2023. Based on MRI findings, patients were categorized into a cerebral infarction group and a non-infarction group. Clinical data, cerebrospinal fluid (CSF) examinations, and imaging features (such as hydrocephalus, cerebral arteritis, and meningeal thickening) were compared between the two groups. Binary logistic regression analysis was used to identify risk factors for acute cerebral infarction in patients with intracranial TB.

**Results:**

A total of 102 patients were included, with 24 in the cerebral infarction group and 78 in the non-infarction group. Male patients accounted for 87.5% in the infarction group and 58.3% in the non-infarction group. Patients with a Glasgow Coma Scale (GCS) score of 3–10 accounted for 45.8% in the infarction group compared to 15.4% in the non-infarction group. The incidence of hydrocephalus, cerebral arteritis, and meningeal thickening was significantly higher in the infarction group (37.5, 54.2, and 79.2%, respectively) compared to the non-infarction group (6.4, 6.4, and 43.6%, respectively) (*p* < 0.05). The parenchymal type of intracranial TB was less frequent in the infarction group (20.8%) than in the non-infarction group (56.4%), while the mixed type was more frequent in the infarction group (62.5%) compared to the non-infarction group (26.9%) (*p* < 0.05). Patients with meningeal thickening involving the cisterns and basal cisterns had a higher risk of cerebral infarction (*p* < 0.05). Multivariate binary logistic regression analysis revealed that male sex (OR = 13.56; 95% CI 1.25–38.30) and cerebral arteritis (OR = 19.32; 95% CI 0.94–37.64) were independent risk factors for cerebral infarction in intracranial TB patients.

**Conclusion:**

Male sex and the presence of cerebral arteritis are independent risk factors for acute cerebral infarction in patients with intracranial tuberculosis.

## Introduction

Intracranial tuberculosis (TB) is a severe form of extrapulmonary TB characterized by non-suppurative inflammation of the brain parenchyma and meninges, resulting from the hematogenous spread of *Mycobacterium tuberculosis* to the central nervous system. It accounts for approximately 1% of all TB cases and 5–10% of extrapulmonary TB cases (1). Intracranial TB carries a high mortality and morbidity rate in both children and adults, with mortality ranging from 15 to 60% (2–4). The diagnosis remains challenging due to the nonspecific early symptoms and limitations of laboratory tests (5), and reliable epidemiological data are still lacking (6). Among survivors, over half are left with neurological sequelae, including cognitive impairment and motor dysfunction (7).

The incidence of cerebral infarction in intracranial TB varies widely, from 15 to 60% (8–10). This discrepancy is likely due to differences in patient populations and the improved detection of infarctions with the increased use of diffusion-weighted imaging (DWI) in MRI (11). Cerebral infarction is a key predictor of long-term neurological damage and an independent risk factor for mortality in intracranial TB patients (12). Some studies have identified risk factors associated with the occurrence of cerebral infarction in intracranial TB. Wasay et al. (12) found that age over 40, hypertension, hyperlipidemia, and diabetes are significant predictors of acute cerebral infarction in this population, while Kalita et al. (13) reported a strong correlation between cerebral infarction and meningitis, hydrocephalus, and hypertension.

Despite these findings, there remains a lack of comprehensive studies, both domestically and internationally, on the risk factors and underlying mechanisms of acute cerebral infarction in intracranial TB. Therefore, this study aims to investigate the clinical and imaging risk factors associated with acute cerebral infarction in patients with intracranial TB, with the goal of improving early recognition and management of this severe complication.

## Methods

### Study design and patients

This retrospective study assembled clinical data and contrast-enhanced MRI results from patients diagnosed with intracranial tuberculosis for the first time, admitted to Hangzhou Red Cross Hospital between January 2020 and March 2023. The collected data included demographic information, comorbidities, onset timing, Glasgow Coma Scale (GCS) score, cerebrospinal fluid (CSF) examination, albumin index, hospital stay duration, discharge outcome, lesion location, enhancement method, and the presence or absence of conditions such as hydrocephalus and cerebral arteritis. Inclusion criteria were as follows: (1) Patients who had been definitively diagnosed or first clinically diagnosed with intracranial tuberculosis; (2) No prior history of cerebrovascular diseases, HIV, central nervous system infections, or tumors; (3) Comprehensive clinical data including head MRI enhancement examination within 3 days of hospitalization. The study protocol was approved by the Ethics Committee of Hangzhou Red Cross Hospital.

The diagnosis of intracranial tuberculosis was based on the criteria established by Marais in 2010 (14). Definitive criteria involved the presence of acid-fast bacilli in the CSF, culture of *Mycobacterium tuberculosis* from CSF, or a positive CSF *M. tuberculosis* nucleic acid amplification test in a patient presenting symptoms or signs indicative of meningitis such as headache, vomiting, neck rigidity, or consciousness disorders. Probable criteria encompassed imaging examination in line with the manifestations of intracranial tuberculosis and a score ≥12, with at least 2 points arising from CSF or cerebral imaging criteria.

### MRI scanning and image analysis

All patients underwent both plain and enhanced brain MRI examination using 3.0 T (Philips Achieva) and 1.5 T (GE Signa Explorer) MR scanners. Transverse T1, T2, T2 fluid attenuated inversion recovery (FLAIR), diffusion-weighted imaging (DWI), and sagittal fat suppression T2 images were acquired (FOV 220, slice thickness 5 mm, interslice gap 1 mm). Enhanced MRI scanning employed Gadolinium gluconate (dose 0.1 mmol/Kg), with sagittal T1 (FOV 240, slice thickness 1 mm, interslice gap 1 mm, matrix 224 × 320) scanned and automatic reconstruction of coronal and cross-sectional images performed.

**Figure 1:**
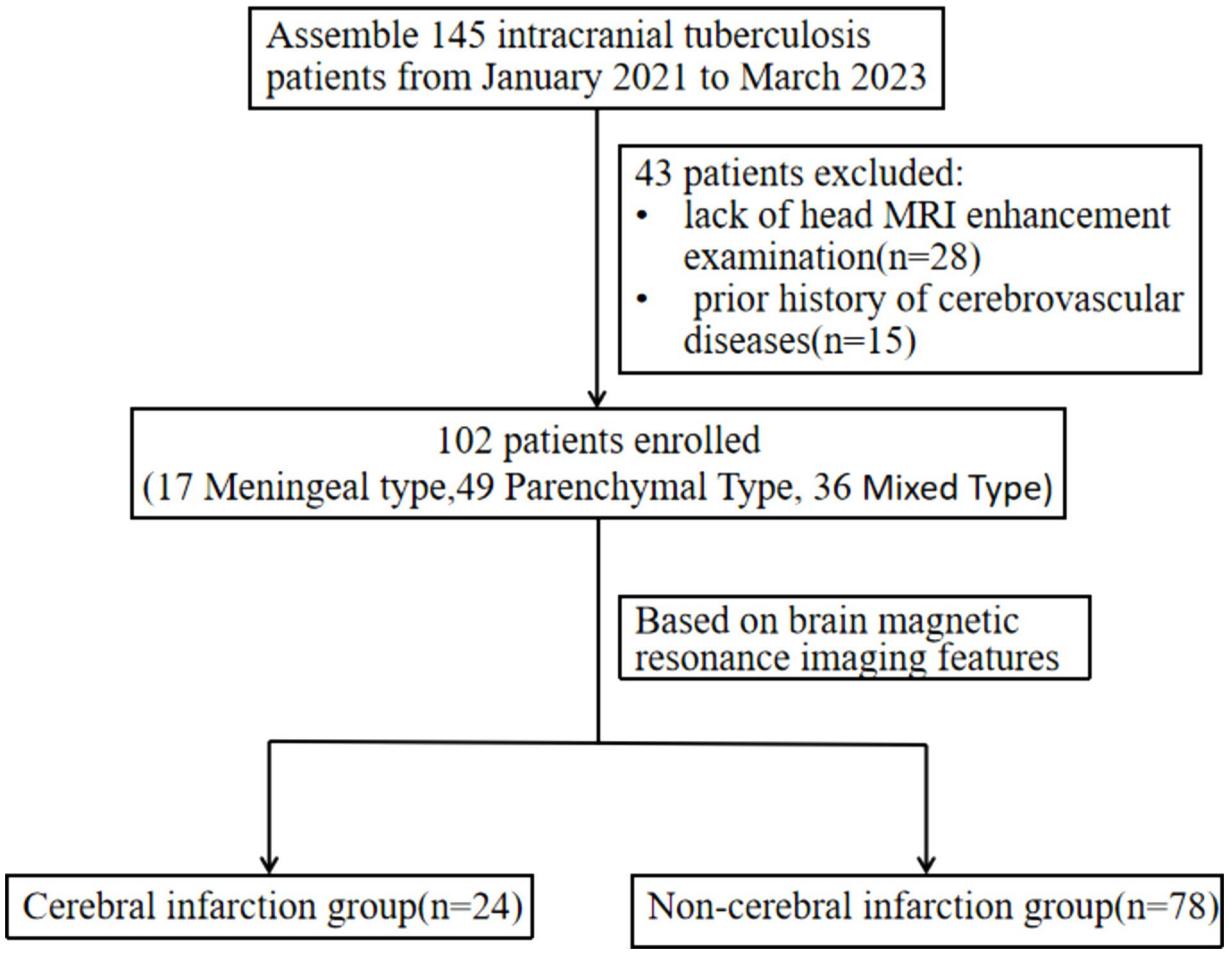
Flow diagram of participants.

Two experienced radiologists identified the lesion location, signal characteristics and enhancement, meningeal thickening, hydrocephalus, acute cerebral infarction and its location, cerebral arteritis, and exudation. Observations were focused on meningeal thickening in areas such as the circular cistern, basal cistern, lateral fissure cistern, cerebral convex meninges, along with meningeal nodules and tuberculosis abscesses. Patients were subsequently categorized into cerebral infarction and non-infarction groups based on imaging findings and classified as meningeal tuberculosis, brain parenchymal tuberculosis, or mixed intracranial tuberculosis according to their imaging types. Meningeal tuberculosis refers to a form of tuberculosis where the lesions affect only the meninges, including the dura mater, pia mater, basal cisterns, and ventricular ependyma. Brain parenchymal tuberculosis refers to a form of tuberculosis where the lesions affect only the brain parenchyma, including tuberculomas, tuberculous granulomas, tuberculous encephalitis, and tuberculous brain abscesses. Mixed intracranial tuberculosis refers to the presence of both meningeal tuberculosis and brain parenchymal tuberculosis in the same case (Figure 1).

### Statistical analysis

All statistical analyses were performed using SPSS (version 27.0) and R software (version 4.4.1). Continuous variables were expressed as mean ± standard deviation (SD) for normally distributed data, and as median (interquartile range, IQR) for non-normally distributed data. Categorical variables were presented as frequencies (percentages). The Shapiro–Wilk test was used to assess the normality of continuous variables. The following statistical methods were applied for group comparisons: (1). Independent samples t-test for normally distributed continuous variables and the Mann–Whitney U test for non-normally distributed data. (2). Chi-square test or Fisher’s exact test for categorical variables, depending on the expected counts. To explore risk factors associated with acute cerebral infarction in patients with intracranial tuberculosis, univariate logistic regression was first conducted. Variables with a *p*-value <0.10 were included in the multivariate binary logistic regression to identify independent risk factors. The results of the logistic regression were presented as odds ratios (OR) with 95% confidence intervals (CI). Due to the relatively small sample size of the study, the bootstrap method (with 1,000 resamples) was applied during multivariate analysis to ensure the robustness and reliability of the results. This technique helped mitigate any potential bias or overfitting caused by the limited sample size. A heatmap was created to visualize the correlation between different types of intracranial tuberculosis and the occurrence of acute cerebral infarction. Additionally, a forest plot was generated from the multivariate logistic regression analysis to display the effect sizes (ORs) of the independent risk factors. All statistical tests were two-tailed, with a *p*-value <0.05 considered statistically significant.

## Results

Our retrospective study included 102 patients diagnosed with intracranial tuberculosis, characterized by a median age of 40.1 ± 18.8 years, comprising 63 males and 39 females. The study subdivided these cases into two groups: the cerebral infarction group, which comprised 24 cases (4 meningeal types, 5 parenchymal types, 15 mixed types), and the non-infarction group, which contained 78 cases (13 meningeal types, 44 parenchymal types, 21 mixed types).

Within the infarction group, 21 males and 3 females were reported, while the non-infarction group included 42 males and 36 females. Notably, the infarction group consisted of a higher proportion of males (87.5% vs. 58.3%, *p* = 0.003). Furthermore, a greater number of patients in the infarction group scored 3–10 on the Glasgow Coma Scale (GCS) (45.8% vs. 15.4%, *p* = 0.002). Comorbidities primarily comprised conventional vascular risk diseases, including hypertension and diabetes. No significant disparity was observed between the two groups regarding age distribution, onset duration, underlying disease, length of hospital stay, cerebrospinal fluid examination results, hypoproteinemia prevalence, and prognosis (*p* > 0.05) (Table 1).

**Table 1:** Comparison of clinical data between cerebral infarction and non-cerebral infarction groups.

Variables	Cerebral infarction group (*n* = 24)	Non-cerebral infarction group (*n* = 78)	*B*	*p*-value
Age				
<18 years	3 (12.5%)	3 (3.8%)	2.458	0.117
18–50 years	14 (58.3%)	56 (71.8%)	1.545	0.214
>50 years	7 (29.2%)	29 (37.2%)	0.516	0.473
Gender				
Male	21 (87.5%)	42 (58.3%)	8.802	0.003*
Comorbidities				
Hypertension	3 (12.5%)	5 (6.4%)	0.932	0.334
Diabetes mellitus	5 (20.8%)	8 (10.3%)	1.828	0.176
GCS score			9.743	0.002*
0–10	11 (45.8%)	12 (15.4%)		
11–15	13 (54.2%)	66 (84.6%)		
Onset time			2.606	0.106
≤3 days	11 (45.8%)	22 (28.2%)		
>3 days	13 (54.2%)	56 (71.8%)		
Cerebrospinal fluid				
Increased white blood cells	10 (41.7%)	40 (51.3%)	0.679	0.410
Elevated protein	11 (45.8%)	41 (52.6%)	0.333	0.564
Reduced glucose	4 (16.7%)	7 (9.0%)	1.118	0.290
Decreased chloride	10 (41.7%)	24 (30.8%)	0.981	0.322
Elevated lactate dehydrogenase	4 (16.7%)	19 (24.4%)	0.622	0.430
Hypoalbuminemia	17 (70.8%)	54 (69.2%)	0.004	0.947
Length of hospital stay (days)	35.0 ± 15.0	30.7 ± 15.6	1.179	0.241
Improvement upon discharge	21 (87.5%)	72 (92.3%)	0.522	0.470

**Figure 2:**
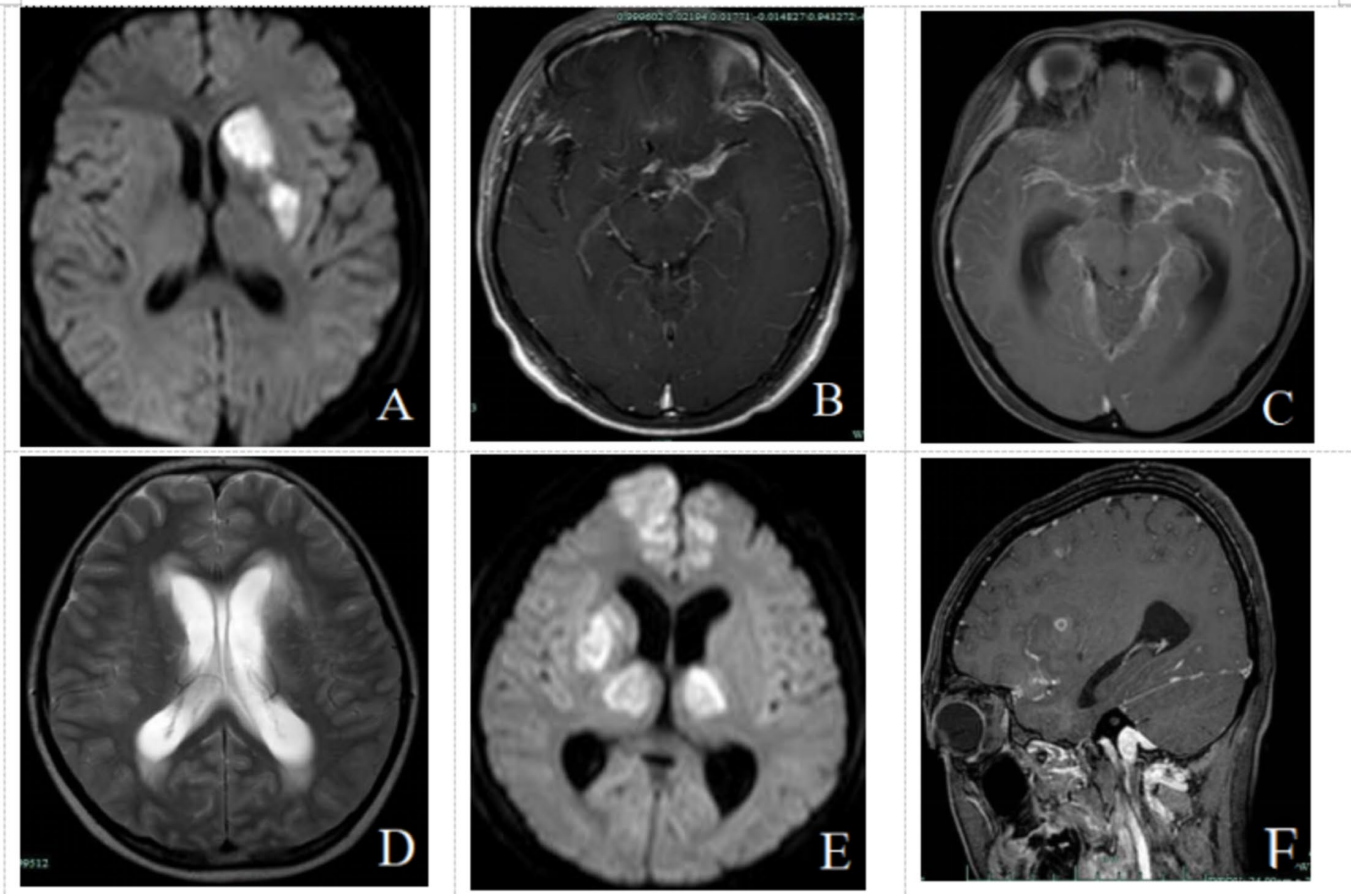
**(A,B)** MRI in a 23-year-old male patient with meningeal type intracranial TB. **(A)** DWI sequence shows acute infarction in the left basal ganglia. **(B)** Enhanced MRI shows M1 segment arteritis of left middle cerebral artery. **(C,D)** MRI in a 44-year-old man with mixed type intracranial TB. **(C)** Enhanced MRI showing changes in Willis ring arteritis. **(D)** T2WI shows Hydrocephalus. **(E,F)** MRI in a 23-year-old woman pregnant with mixed type intracranial TB. **(E)** DWI sequence shows multiple acute infarction in bilateral frontal lobes, right basal ganglia, and bilateral thalamus. **(F)** Sagittal enhanced MRI showing multiple lesions in the brain parenchyma and meninges.

Increased meningeal thickening was observed in the infarction group compared to the non-infarction group (79.2% vs. 43.6%, *p* = 0.002). Furthermore, the infarction group demonstrated a higher prevalence of hydrocephalus (37.5% vs. 6.4%, *p* = 0.000), cerebral arteritis (54.2% vs. 6.4%, p = 0.000), and meningeal thickening (79.2% vs. 43.6%, *p* = 0.002). In examining the association between the location of meningeal thickening and cerebral infarction, we discovered that ambient and basal cistern meningeal thickening tended to be associated with a higher risk of cerebral infarction (Figure 2). No significant variance was detected in the incidence of tuberculous brain abscess and meningeal nodules between the two groups (Table 2).

**Table 2:** Comparison of MRI imaging findings between cerebral infarction and non-cerebral infarction groups.

Variables	Cerebral infarction group (*n* = 24)	Non-cerebral infarction group (*n* = 78)	*B*	*p*-value
Meningeal nodules	1 (4.2%)	12 (15.4%)	2.056	0.152
Tuberculous brain abscess	1 (4.2%)	5 (6.4%)	0.165	0.684
Hydrocephalus	9 (37.5%)	5 (6.4%)	14.834	0.000*
Cerebral arteritis	13 (54.2%)	5 (6.4%)	28.519	0.000*
Meningeal thickening	19 (79.2%)	34 (43.6%)	9.306	0.002*
Location of meningeal thickening				
Perimesencephalic/cisternal meninges	14 (58.3%)	12 (15.4%)	17.825	0.000*
Lateral sulcus meninges	9 (37.5%)	17 (21.8%)	2.383	0.123
Cerebral convexity meninges	7 (29.2%)	15 (19.2%)	1.071	0.301
Other meningeal sites	2 (8.3%)	11 (14.1%)	0.544	0.461

Other meningeal sites mainly include the interhemispheric fissure cistern, the cerebellomedullary cistern, and the meningeal regions around the choroid plexus of the lateral ventricles.

**Figure 3:**
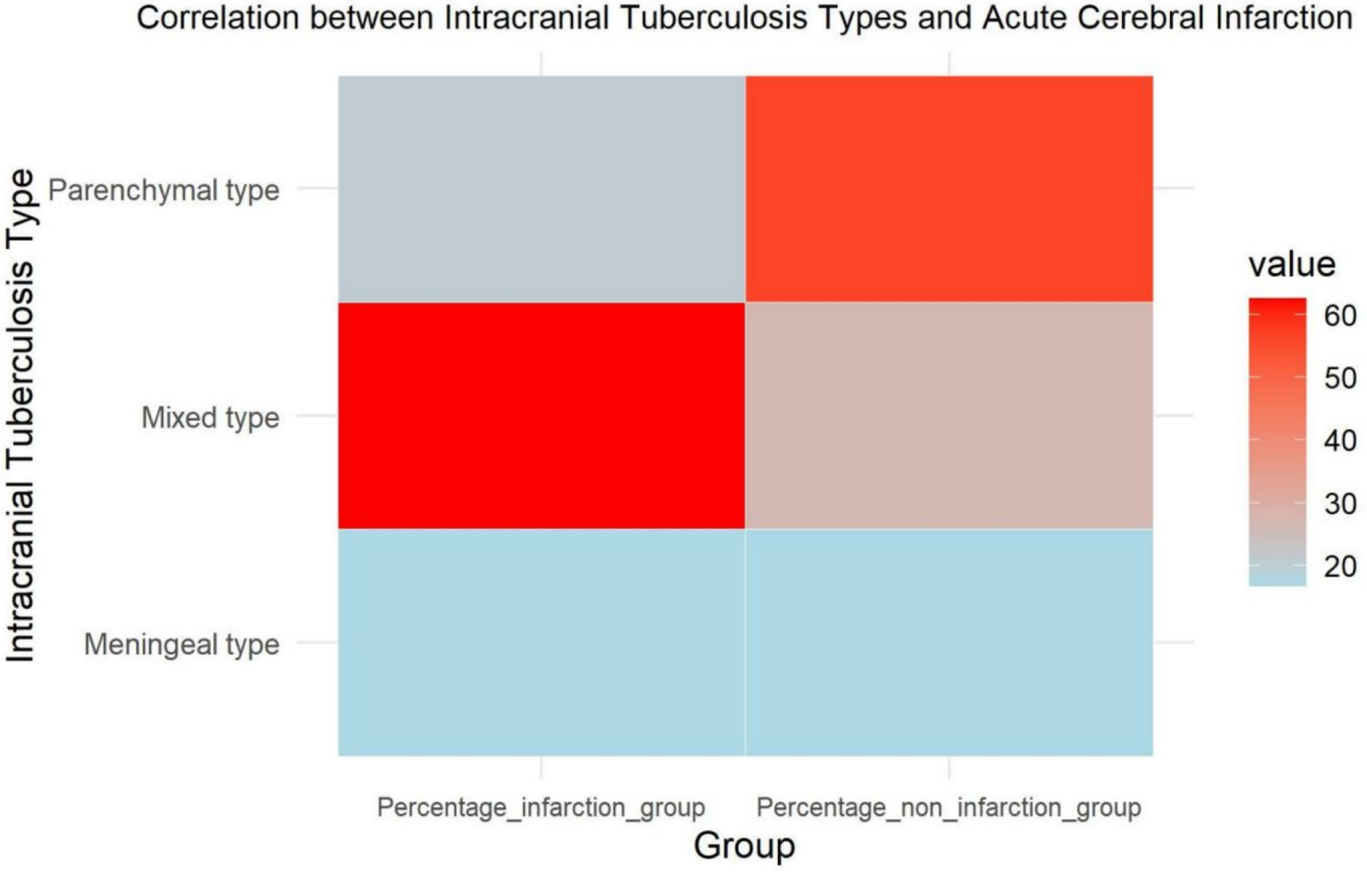
Heatmap of the correlation between different types of intracranial tuberculosis and acute cerebral infarction.

To further analyze the correlation between different types of intracranial tuberculosis and cerebral infarction, we observed equal proportions of meningeal tuberculosis (16.7%) in two groups. Parenchymal tuberculosis was reported in 20.8% of the infarction group and 56.4% of the non-infarction group, while mixed tuberculosis accounted for 62.5 and 26.9%, respectively. There was no significant difference between the two groups regarding the proportion of meningeal tuberculosis. Among patients with parenchymal tuberculosis, fewer cases of cerebral infarction were noted, while this condition was more prevalent among those with mixed intracranial tuberculosis (Table 3; Figure 3).

**Table 3:** Analysis of the correlation between different types of intracranial tuberculosis and acute cerebral infarction.

Variables	Cerebral infarction group (*n* = 24)	Non-cerebral infarction group (*n* = 78)	*B*	*p*-value
Meningeal type	4 (16.7%)	13 (16.7%)	0.000	1.000
Parenchymal type	5 (20.8%)	44 (56.4%)	9.306	0.002
Mixed type	15 (62.5%)	21 (26.9%)	10.172	0.001

Binary logistic regression analysis was employed to delineate the risk factors of cerebral infarction, encompassing gender, hydrocephalus, cerebral arteritis, Glasgow Coma Scale (GCS) score. The analysis revealed that male gender (OR = 13.56; 95% CI 1.25–38.30) and cerebral arteritis (OR = 19.32; 95% CI 0.94–37.64) were independent risk factors for cerebral infarction. (Table 4).

**Table 4:** Multivariate binary logistic regression analysis.

Variables	OR (95%CI)	*p*-Value
Male	13.56 (1.25–38.30)	0.003
Cerebral arteritis	19.32 (0.94–37.64)	0.004

## Discussion

This retrospective study of intracranial tuberculosis and ischemic stroke showed male sex and cerebral arteritis are independent risk factors for acute cerebral infarction in patients with intracranial tuberculosis. *Mycobacterium tuberculosis* can invade the central nervous system (CNS) either by direct infiltration, migration to vascular endothelial cells, or via macrophages and neutrophils, inducing a range of cerebrovascular pathologies (15). The most common pathological changes in the vascular walls of intracranial TB patients include infiltration, proliferation, and necrosis, which often occur simultaneously (15). The reported incidence of cerebral infarction in intracranial TB patients varies greatly across studies. In pediatric populations, the incidence is considerably higher than in adults. For example, Solomons et al. (16) reported that up to 71.5% of pediatric patients with intracranial TB develop cerebral infarction. In our study, the incidence was approximately 23.5%, and this significant difference may be attributed to the age of the study population. Further research is required to explore the underlying reasons.

Factors such as age and comorbidities did not show statistically significant differences between the cerebral infarction and non-infarction groups in our study, which contrasts with the findings of Wasay et al. (12). However, we observed that the incidence of cerebral infarction was significantly higher in males than females, with a peak incidence in middle-aged individuals, aligning with the epidemiology of cerebral infarction in the general population. A large-scale study from Guangdong, China, found that 67.1% of young stroke patients were male (17), and in Western countries, males also account for a slightly higher proportion of stroke cases (18). The mechanisms underlying this gender difference remain unclear but may be related to higher rates of smoking, alcohol consumption, and other vascular risk factors among males (19). Additionally, estrogen levels, particularly 17-β estradiol, which has anti-inflammatory properties and supports cholesterol and lipoprotein metabolism, may offer protective effects in younger women (20). Previous studies have identified traditional cerebrovascular risk factors, such as advanced age, dyslipidemia, hypertension, and hyperglycemia, as well as elevated white blood cell levels in cerebrospinal fluid (CSF), TB disease duration, meningitis, hydrocephalus, and cranial nerve palsy as factors associated with the risk of cerebral infarction in intracranial TB (10, 12, 13). In our study, the proportion of male patients with intracranial TB and cerebral infarction reached 87.5%. Besides the potential influence of traditional vascular risk factors, it remains to be seen whether gender differences in immune responses to *M. tuberculosis* play a role in the increased susceptibility to cerebral infarction, warranting further investigation.

In our study, intracranial TB patients with cerebral infarction were more likely to experience varying degrees of consciousness disturbances. This may be due to early-stage TB exudates causing inflammatory infiltration of blood vessels, leading to ischemic vasospasm of small arteries and subsequent clinical symptoms (15). At this stage, cerebral infarctions are often secondary to vasospasm and vasculitis. As the disease progresses, the accumulation of inflammatory infiltrates in the vessel walls leads to proliferative endarteritis, and in some cases, atherosclerosis, vessel stenosis, or occlusion, further promoting cerebral infarction through hemodynamic hypoperfusion. In the study by Kalita et al. (13), among 54 patients with intracranial tuberculosis who developed cerebral infarction, 13 cases occurred within 3 months of receiving treatment. And in our study, there was one case in which a new infarction was found during follow-up, despite improvement in the original infarcted area. Therefore, for patients with intracranial tuberculosis, timely and effective treatment may help effectively suppress vasospasm, vasculitis, intimal hyperplasia, and even thrombosis (21), which is crucial for improving prognosis.

We also found a correlation between the imaging type of intracranial TB and the occurrence of cerebral infarction (22). Parenchymal TB was associated with a lower incidence of cerebral infarction, while mixed-type TB was more likely to result in infarction. Although meningeal thickening was found to be associated with a higher risk of cerebral infarction, there was no statistically significant difference in the likelihood of cerebral infarction between meningeal-type TB cases, which may be due to the smaller number of cases with meningeal-only involvement, typically observed in early-stage disease. When meningeal thickening involved the perimesencephalic and basal cisterns, the risk of cerebral infarction was significantly higher, consistent with findings from Zhang et al. (10). After *M. tuberculosis* invades the CNS, it initially deposits on the pia mater or ependyma, triggering immune responses that result in meningeal edema and gelatinous exudation. This exudate accumulates primarily in the basal cisterns, encasing the vessels and nerves, and may lead to cranial nerve palsy (23). The circle of Willis is especially vulnerable to infiltration by TB exudates, leading to cerebral arteritis, which appears on enhanced MRI as thickened, irregular arterial walls with luminal narrowing. Widespread involvement of the basal arteries is often accompanied by hydrocephalus (24). This explains why the incidence of cerebral arteritis and hydrocephalus was significantly higher in the cerebral infarction group compared to the non-infarction group. In recent years, with the continuous development and innovation of MRI equipment and scanning technologies, MRI vessel wall imaging techniques have gradually matured (25). These advancements allow for clearer and more intuitive detection of arterial wall lesions, and may potentially become a new research direction for intracranial tuberculosis complicated by cerebral infarction.

This study has several limitations, including the small sample size of patients with cerebral infarction, which precluded detailed analysis of infarction locations. Future research should focus on expanding the cohort and conducting long-term follow-up to investigate the impact of cerebral infarction on the prognosis of intracranial TB patients.

## Conclusion

Patients with cerebral infarction secondary to intracranial tuberculosis are relatively common. Our regression analysis identified male sex and the presence of cerebral arteritis as independent risk factors for acute cerebral infarction in patients with intracranial TB. Besides, the likelihood of cerebral infarction varies among different types of intracranial TB. These findings highlight the need for further investigation into the pathophysiological differences between TB types and their relationship with cerebral infarction.

## Data Availability

The original contributions presented in the study are included in the article/supplementary material, further inquiries can be directed to the corresponding author.
